# REEP4 as Potential Biomarker Associated with Predictive Prognosis and Immune Response in Kidney Clear Cell Carcinoma

**DOI:** 10.7150/jca.96135

**Published:** 2024-06-03

**Authors:** Zixuan Chen, Xing Jia, Min Liu

**Affiliations:** Department of Urology, Tongren Hospital Shanghai Jiao Tong University School of Medicine, Shanghai, 200336, China.

**Keywords:** REEP4, KIRC, Immunity, Prognosis, Biomarker

## Abstract

Kidney clear cell carcinoma (KIRC) commonly presents with metastases upon diagnosis, highlighting the critical need to identify more precise biomarkers for early detection, intervention, and personalized treatment. Although The REEP family has been investigated in cancer development, the specific relationship between REEP4 and cancer remains unclear. In our study, we employed bioinformatics analysis and conducted fundamental experiments to evaluate the potential of REEP4 as a biomarker for predicting the prognosis and therapeutic efficacy of KIRC. Comparing KIRC tumor tissues to normal tissues, we observed a significant upregulation in REEP4 expression, with higher levels of REEP4 correlating positively with tumor malignancy. Further COX regression analysis, as well as single and multifactorial analyses, confirmed that high REEP4 expression indicated lower survival rates in KIRC. Gene function analysis also identified associations between REEP4 and critical pathways such as the cell cycle, along with its involvement in protein binding. Furthermore, our investigation of the immune response suggests that a favorable immunotherapeutic response is linked to a reduction in REEP4 expression. Subsequently, we conducted in *vitro* experiments to confirm the overexpression of REEP4 in KIRC tumor tissues and renal cancer cells. In summary, our study revealed a close association between REEP4 expression and KIRC, emphasizing its correlation with prognosis and the immune response. These findings suggest that REEP4 is a potential biomarker for KIRC.

## Introduction

Kidney cancer is one of the most common cancers and accounts for approximately 2% of all cancer diagnoses and cancer-related deaths worldwide. The vast majority of kidney cancers (over 90%) are renal cell carcinomas (RCC), which constitute a diverse group of renal tubular epithelial cells 1. RCC encompasses several major histological subtypes, with clear cell RCC (ccRCC), papillary RCC (pRCC), and chromophobe RCC (chRCC) being the most prevalent subtypes. Among these, ccRCC, also known as KIRC, is the most frequently encountered type 2. Typically, KIRC responds well to tyrosine kinase and immune checkpoint inhibitors 3. However, its high metastasis and drug resistance present formidable clinical challenge 4. Consequently, identifying a reliable biomarker for prognostication and evaluation of clinical interventions for KIRC is imperative.

REEP4, a member of the receptor expression-enhancing protein (REEPs) family, plays a pivotal role in the structure and function of the endoplasmic reticulum (ER) 5. In addition to its localization in the cytoplasmic ER, REEP4 is also associated with the nuclear envelope (NE). This enables it to link the high-curvature ER to the ELYS-based nuclear pore complex (NPC) seed, thereby promoting NPC biogenesis during late mitosis 6. To date, REEP4 has been linked to aneuploid pregnancies 7, a disease intricately connected with mitosis. Although other members of REEP have been found to be associated with breast cancer 8, hepatocellular carcinoma 9, and lung cancer 10, no reports have addressed the correlation between REEP4 and cancer. Therefore, it is crucial to investigate whether REEP4 can function as a cancer marker.

**Figure 1:**
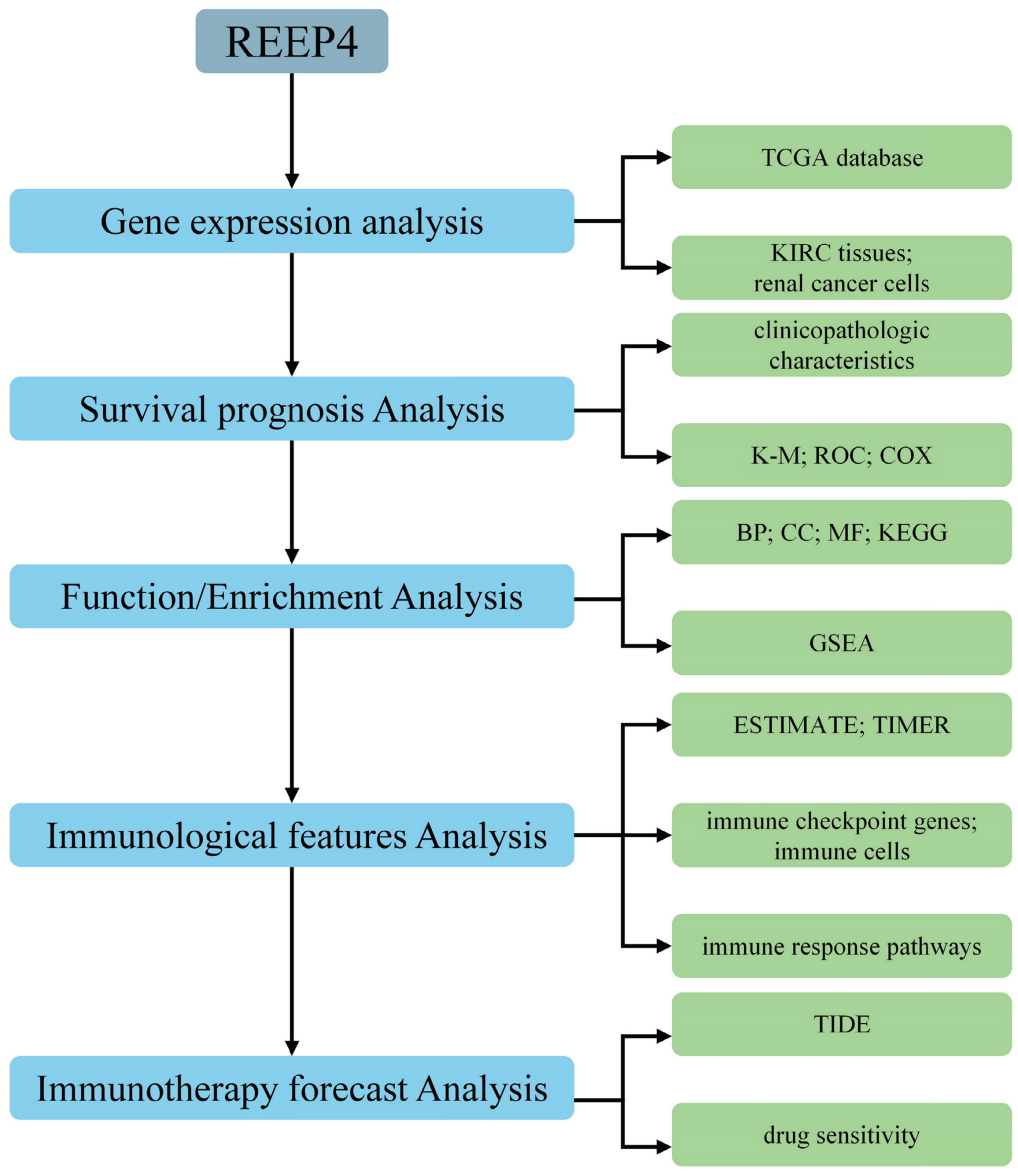
Flowchart for analyzing REEP4 as potential biomarker for KIRC.

Hence, we focused on REEP4 and conducted a comprehensive analysis of its expression profile, biological significance, and immunotherapeutic potential in KIRC through diverse bioinformatics analyses and experiments. This study aimed to elucidate the potential of REEP4 as a biomarker for assessing the prognosis and therapeutic effectiveness of KIRC (Figure 1).

## Material and Methods

### Sample Collection

Cancerous and adjacent non-cancerous tissue samples from ten KIRC patients were collected from Tongren Hospital, Shanghai Jiao Tong University School of Medicine. All patients signed a medical informed consent document after being informed of the study, which was approved by the Ethics Committee of Tongren Hospital, Shanghai Jiao Tong University School of Medicine. Inclusion criteria were diagnosis of KIRC before surgery, confirmation by histopathology after surgery, and no history of other malignancies. Exclusion criteria were patients who received preoperative adjuvant therapy, such as radiotherapy or chemotherapy. And the clinical information of the patients is shown in Table S1.

### Cell lines

Human RCC cell lines 786-O, A498, Caki-1, and HK-2 were acquired from the Cell Bank of the Chinese Academy of Science. The cells were cultured in RPMI 1640 medium (BasalMedia, China) or Dulbecco's modified essential medium (Gibco, Grand Island, NY, USA) supplemented with 1% P/S (Gibco, Grand Island, NY, USA) and 10% fetal bovine serum (Gibco, Grand Island, NY, USA). The cultures were maintained at 37 °C in a humidified incubator with 5% CO_2_.

### Gene expression analysis

The TIMER technique was used to examine the expression pattern of REEP4 in TCGA cohorts of diverse cancers and typical tissues (https://cistrome.shinyapps.io/timer/) 11. Additionally, 20 normal tissue samples and 20 KIRC samples were obtained from the GEO dataset (GSE213324) to verify the upregulation of REEP4 in tumor tissues using GEO2R (https://www.ncbi.nlm.nih.gov/geo/geo2r/). The differential expression levels of REEP4 in normal and KIRC tissues were assessed using t-tests, and the results were analyzed and visualized using GraphPad Prism. Clinical and follow-up data from TCGA database were downloaded from GDC (https://portal.gdc.cancer.gov/) for subsequent analysis.

### Gene prognostic value assessment

Kaplan-Meier(K-M) survival curves and Receiver Operating Characteristic (ROC) curves were used to evaluate the prognostic significance of REEP4 expression in KIRC. These analyses were performed using data from the TCGA database and graphically represented using the R language package, including “ggplot2”, “ggpubr”, “survminer” and “survival” for K-M, and “survivalROC” for ROC.

To analyze the relationship between REEP4 expression levels and clinicopathological characteristics, we utilized TCGA data, incorporating factors such as age, sex, ethnicity, WHO stage, clinical T stage, clinical N stage, and clinical M stage. The results were visualized using R language, with P-values obtained through t-tests or One-way ANOVA using GraphPad Prism. We also conducted univariate and multivariate COX analyses using SPSS software to obtain p-values and 95.0% CI for Exp(B), followed by visualization using GraphPad Prism.

### Gene function analysis

The most relevant genes of REEP4 were uploaded to The Database for Annotation, Visualization and Integrated Discovery website (https://david.ncifcrf.gov/) 12. We selected the “official gene symbol” for Identifier and “Homo sapiens” for species. Gene Ontology (GO) and Kyoto Encyclopedia of Genes and Genomes (KEGG) pathway analyses were performed. Finally, we selected the top six results based on P values (P<0.05) and visualized them using R language. Then, using gene set enrichment analysis (GSEA), target genes were analyzed using the database to annotate, visualize, and integrate discovery to find potential pathways (http://www.kegg.jp/). Nominal FDR < 25% and P value < 0.05 were deemed statistically significant.

### Immunological correlation analysis

The ESTIMATE tool was employed to acquire “StromalScore”, “ImmuneScore”, and “ESTIMATEScore” for assessing the connection between REEP4 and the KIRC tumor microenvironment. Additionally, TIMER was used to evaluate the correlation between REEP4 expression and immune cell infiltration, including CD8+ T cells, B cells, dendritic cells, CD4+ T cells, macrophages, and neutrophils. Relevant data were calculated using Sangerbox 3.0 (http://sangerbox.com/). Subsequently, we analyzed the influence of REEP4 expression on immune pathways using GSVA.

### Immunotherapy forecast analysis

The immunophenoscore (IPS) was obtained from the TCIA database(https://tcia.at/) 13. We also calculated scores for TIDE, Dysfunction, and Exclusion using the Tumor Immune Dysfunction and Exclusion (TIDE) tool (http://tide.dfci.harvard.edu/) 14. KIRC patients from TCGA database were divided into high and low expression groups based on REEP4 expression levels, and the Spearman correlation coefficient was used to predict the relationship between REEP4 expression and KIRC immunotherapy. Correlation analysis between REEP4 expression and drug sensitivity was performed using the CellMiner dataset (http://discover.nci.nih.gov/cellminer/). Data processing and graphing were carried out through R/Bioconductor package of the “ggpubr”, “limma” and “impute”.

### Western blot

Cell lysates were obtained by treating the cells with RIPA lysis buffer (Beyotime Biotechnology, Shanghai, China) supplemented with PMSF and protease inhibitors (Beyotime Biotechnology). Protein concentration was determined using a BCA Protein Assay Kit (K4104, APExBIO, Houston, USA). Proteins were separated using SDS-PAGE and then transferred onto PVDF membranes (Millipore Corporation, USA). Membranes were blocked with 5% skim milk for 1 h at room temperature. Rabbit polyclonal anti-REEP4 (Proteintech, USA, 1:1,000) and anti-β-Tubulin (Abcam, UK, 1:5,000) were used to incubate the membranes overnight at 4°C. The membranes were washed three times with TBST and probed with goat anti-rabbit IgG (H + L) and HRP conjugate (Abcam, UK, 1:10,000) for 2 h at room temperature. The blots were then washed again with TBST. To visualize the blots, a Tanon-5200 chemiluminescence imaging system (Tanon Science & Technology, Shanghai, China) was used, and grayscale analysis was performed using Image-J software.

### Real-time PCR (RT-PCR) analysis

Total RNA was extracted from cancerous and adjacent non-cancerous tissues of KIRC patients by treating them with RNAiso Plus (TaKaRa, Japan). Reverse transcription was carried out using HiScript III RT SuperMix for qPCR (+gDNA wiper) (Vazyme, China) to obtain cDNA, followed by PT-PCR using ChamQ Universal SYBR qPCR Master Mix (Vazyme, China) and a CFX96 Touch Real-Time PCR Detection System (Bio-Rad). Statistical analysis of the transcript levels of the actin gene was used for normalization. Statistical analysis and visualization were performed using the GraphPad Prism software. REEP4 primer: 5'-GCAGCAGAGATCGTTACAGAC-3' (forward) and 5'-CCCTTGGTGTAGGGTGAGA-3' (reverse).

## Results

### Expression Level of REEP4 is upregulated in KIRC

**Figure 2:**
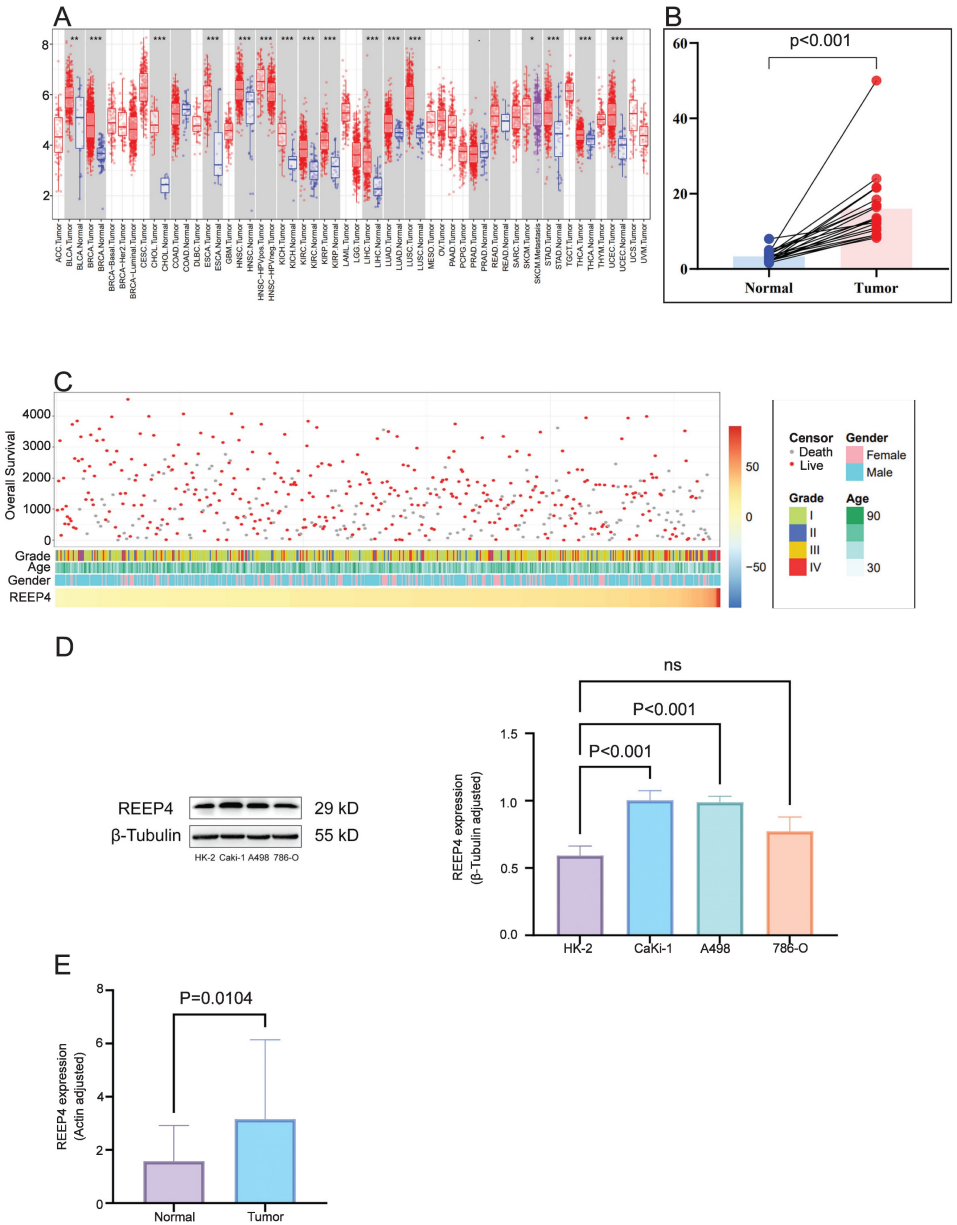
**A** Pan-cancer analysis of the expression of REEP4 in TCGA database (P-value Significant Codes: 0 ≤ *** < 0.001 ≤ ** < 0.01 ≤ * < 0.05 ≤. < 0.1); **B** expression levels of the REEP4 between KIRC tissues and their corresponding normal kidney tissue in GEO dataset; **C** The landscape of REEP4-related clinicopathological features of KIRC in the TCGA database. **D** REEP4 expression in renal cancer cells was determined by Western Blot; **E** RT-PCR analysis of REEP4 expression in KIRC tissues.

In pan-cancer analysis, compared to normal tissues, REEP4 expression was significantly upregulated in various cancers, including breast invasive carcinoma (BRCA), cholangiocarcinoma (CHOL), liver hepatocellular carcinoma (LIHC), and KIRC (P<0.001) (Figure 2A). Utilizing GEO data (GSE213324), which includes 40 samples (one sample lacking corresponding normal tissue data was excluded), we revealed a substantial increase in REEP4 expression in KIRC tissues compared to their corresponding normal kidney tissue (Figure 2B). Furthermore, an extensive examination of TCGA database, which consisted of 529 KIRC samples, demonstrated distinctive clinical and pathological characteristics among patients exhibiting varying levels of REEP4 expression (Figure 2C). And the detailed clinic parameters of the KIRC patients is shown in Table S2.

To enhance the reliability of bioinformatics analysis, validation of KIRC tissues and renal cancer cells was conducted. Western blot results revealed significantly higher expression of REEP4 in A498 and Caki-1 cells than in HK-2 cells (Figure 2D). Consistent with these findings, RT-PCR further confirmed the elevated expression of REEP4 in KIRC tumor tissues (Figure 2E). These results underscore the significance of REEP4 expression levels in KIRC.

### REEP4 is a prognostic factor in KIRC

**Figure 3:**
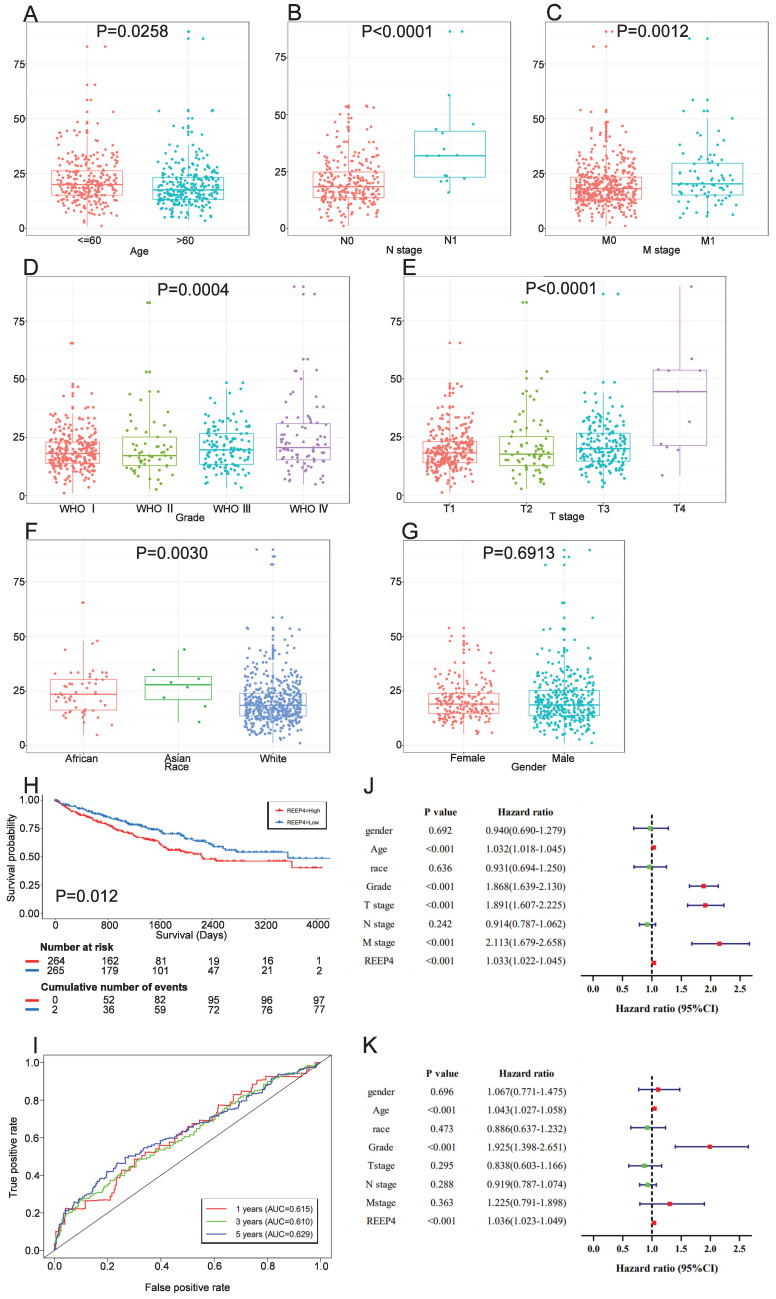
Association between REEP4 expression and clinicopathologic characteristics, including **A** age, **B** N stage, **C** M stage, **D** Grade, **E** T stage, **F** Race, **G** Gender.** H** Kaplan Meier curves of REEP4 in KIRC; **I** ROC curves of REEP4 in KIRC; **J** Univariate cox regression analysis; **K** multivariate cox regression analysis.

To attain a more comprehensive understanding of how REEP4 contributes to KIRC progression, we analyzed REEP4 expression levels in patients with various clinicopathological characteristics. Our data analysis indicated a significant upregulation of REEP4 in advanced tumors, as compared to early tumors, as well as in age, WHO stage, T stage, N stage, and M stage (Figure 3A-E). Moreover, a comparative assessment of REEP4 expression levels among different racial groups, including Caucasians, Asians, and Africans, suggested a correlation between Race and REEP4 expression in One-way ANOVA (P=0.003) (Figure 3F). However, there were no notable disparities in REEP4 expression between sexes (Figure 3G).

Patients were divided into high and low expression groups based on the median REEP4 expression among data from the TCGA database. These groups were then subjected to K-M survival analysis, which showed a significantly lower survival probability for individuals in the high REEP4 expression group than those in the low expression group (P=0.00057) (Figure 3H). Subsequently, ROC curves were generated based on the one-year, three-year, and five-year survival rates. The calculated Area Under Curve (AUC) values were 0.615, 0.610, and 0.629, respectively, indicating moderate predictive accuracy. (Figure 3I).

To further assess the prognostic potential of REEP4, we examined clinicopathological factors using univariate and multivariate COX analyses. Univariate Cox analyses revealed strong correlations between REEP4 expression, WHO grade, T staging, M staging, age, and overall survival (OS) in KIRC (all HR>1, p<0.001) (Figure 3J). Multifactorial Cox regression analysis demonstrated a significant association between high REEP4 expression and reduced OS (HR =1.036; p<0.001). Additionally, both age and WHO grade were associated with OS in the multifactorial COX analysis (Figure 3K). These findings imply that REEP4 serves as an independent prognostic factor, with higher expression levels indicating poorer patient survival. Detailed results are displayed in the Supporting Information (Table S3).

### Biological functions of REEP4 in KIRC

**Figure 4:**
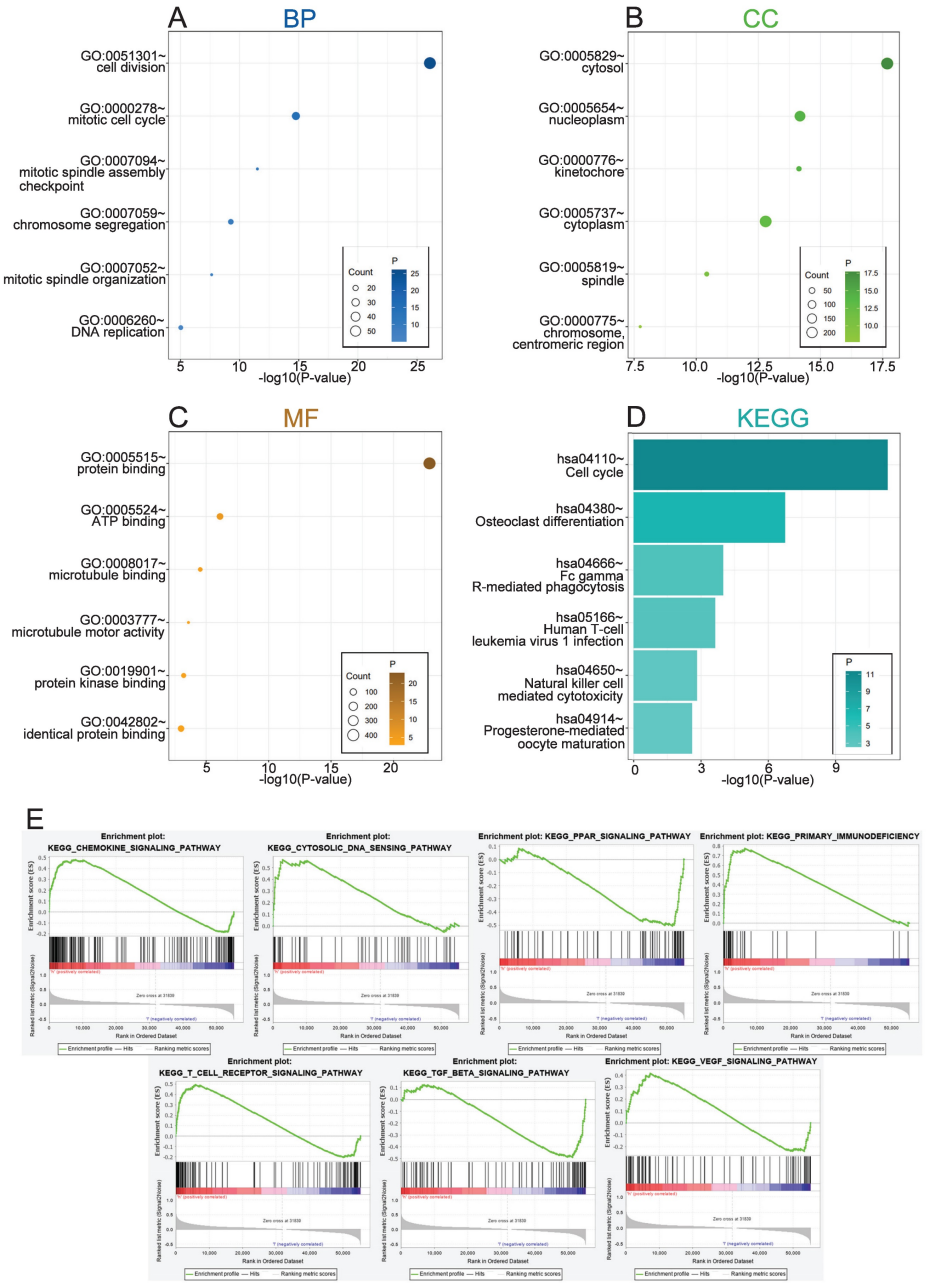
**A** Biological process related to REEP4 in KIRC; **B** cellular components related to REEP4 in KIRC; **C** molecular functions related to REEP4 in KIRC; **D** KEGG pathway analysis of REEP4 in KIRC; **E** Gene sets enrichment analysis of REEP4 mRNA expression in the KIRC cohort.

To explore the biological functions related to REEP4, the 500 genes most related to REEP4 were screened by Pearson correlation analysis (|R| > .5, P < .05) in the TCGA database (Table S4). GO and KEGG analyses were performed based on the above gene sets. We conducted a comprehensive analysis of the function of REEP4, focusing on biological processes (BP), cellular components (CC), molecular functions (MF), and signaling pathways. Our findings revealed that the biological processes associated with REEP4 are mainly related to cell division, mitotic cell cycle, mitotic spindle assembly checkpoint, chromosome segregation, mitotic spindle organization, positive regulation of T cell proliferation and DNA repair (Figure 4A). Additionally, REEP4 was significantly present in cellular components such as the cytosol and nucleoplasm, highlighting its involvement in essential cellular activities (Figure 4B). In terms of molecular function, REEP4 is primarily involved in binding processes, including protein binding, ATP binding, microtubule binding, protein kinase binding, and identical protein binding (Figure 4C). As for the signaling pathways, REEP4 demonstrated a substantial influence on pathways including the cell cycle, osteoclast differentiation, Fc gamma R-mediated phagocytosis, and Human T-cell leukemia virus 1 infection (Figure 4D). These results strongly suggest that REEP4 plays a key role in KIRC development, particularly through its regulatory impact on the cell cycle. GSEA was performed to investigate potential signaling pathways in KIRC patients with high REEP4 expression 15. According to the normalized enrichment score (NES)|> 1.5 and p value < 0.05, several significantly enriched signaling pathways were selected (Figure 4E and Table S5). The results indicated that signaling pathways, including Chemokine, Cytosolic DNA, PPAR, Primary immunodeficiency, T cell receptor, TGF-β, and VEGF, were differentially enriched in high or low REEP4 expression phenotypes.

### Immunological features of REEP4 in KIRC

**Figure 5:**
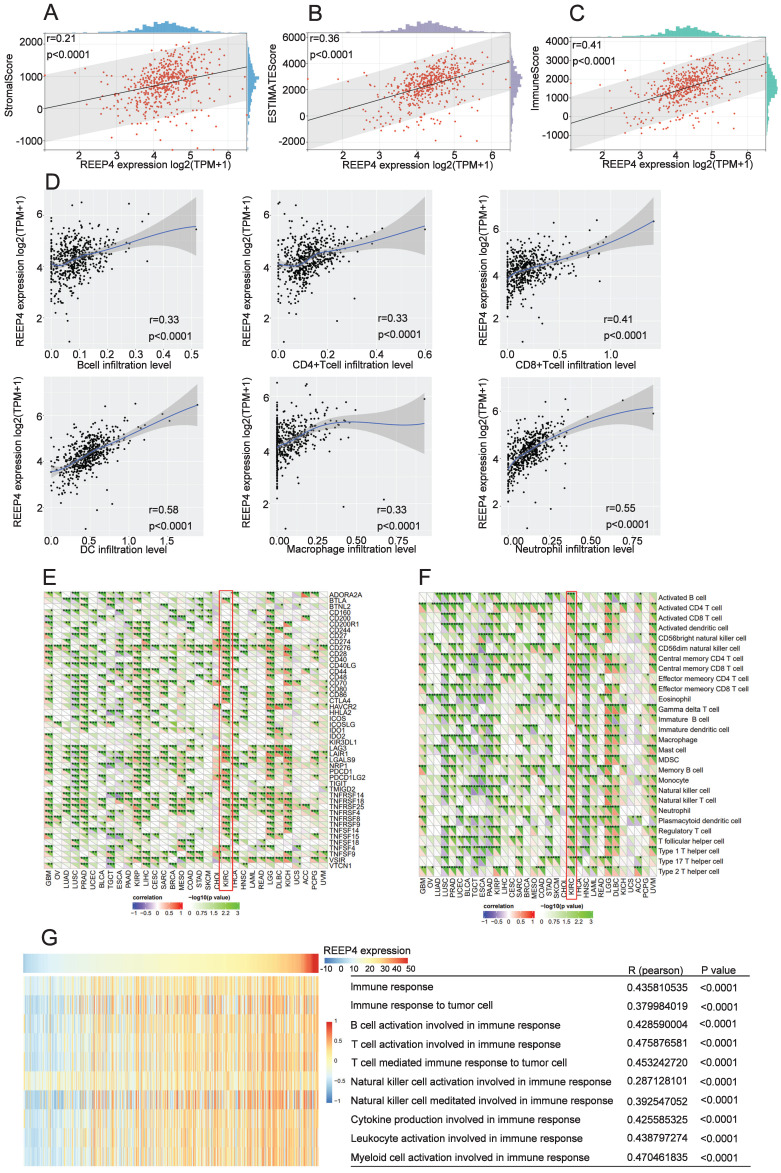
**A** REEP4 expression with the StromalScore; **B** REEP4 expression with the ESTIMATEScore; **C** REEP4 expression with the ImmuneScore; **D** REEP4 expression with immune cells infiltration; Correlation analysis of REEP4 expression and **E** immune checkpoint genes; **F** immune cells; **G** The impact of REEP4 expression on the immune response pathways.

The tumor environment, a complex environment comprising numerous cell types and extracellular components, plays a pivotal role in tumor development, treatment response, and prognosis 16. Upon scrutinizing the impact of REEP4 within the KIRC tumor microenvironment using the ESTIMATE tool, our findings indicated a positive correlation between the expression level of REEP4 and the StromalScore (r=0.21), ESTIMATEScore (r=0.36), and ImmuneScore (r=0.41), and the p-values were all <0.0001(Figure 5A-C). Furthermore, our investigation of tumor cell infiltration using TIMER demonstrated that the upregulation of REEP4 was positively correlated with B cells (r=0.33), CD4+ T cells (r=0.33), CD8+ T cells (r=0.41), DC (r=0.58), macrophages (r=0.33), and neutrophils (r=0.55). All of these associations exhibited p-values <0.0001(Figure 5D). To study the potential targets of KIRC immunotherapy, mRNA sequencing data of KIRC were utilized to assess the association between REPP4 and the acknowledged immune checkpoint genes. This suggests that REPP4 expression is strongly associated with relevant checkpoint genes, such as BTLA, CD244, CD27, CD276, CD48, CD80 and so on (Figure. 5E), as well as immune cells such as activated CD4 T cells, effector memory CD4 T cells, central memory CD4 T cells, and regulatory T cells in the TCGA-KIRC dataset (P < 0.05) (Figure. 5F). Therefore, we evaluated the impact of REEP4 expression on various immune response pathways involving B cells, T cells, natural killer cells, cytokines, leukocytes, and myeloid cells using GSVA (Figure 5G). The results demonstrated a positive correlation between REEP4 expression and all the aforementioned immune functions.

### Impact of REEP4 on immunotherapy in KIRC

**Figure 6:**
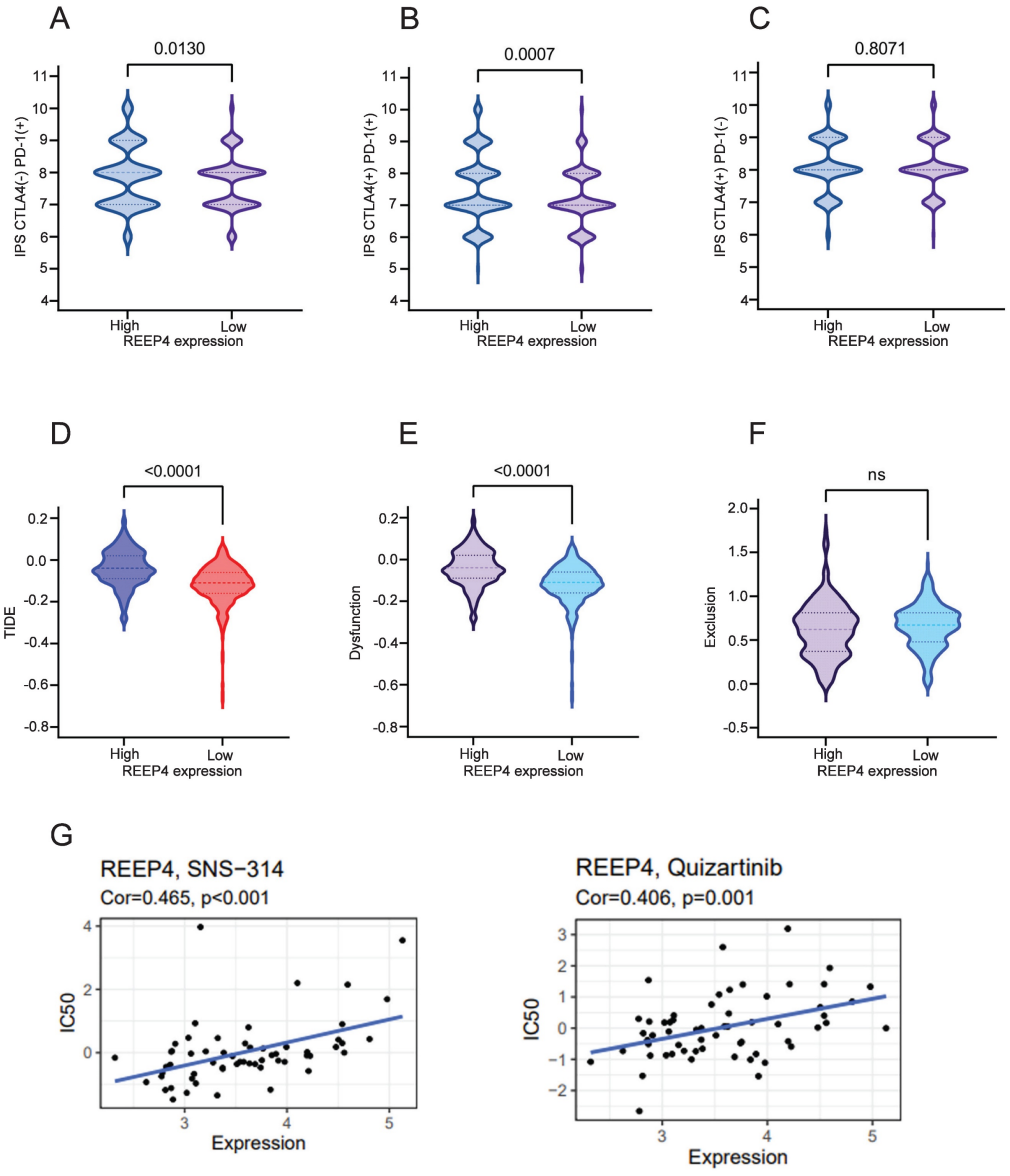
**A** Immune responses of REEP4 to PD-1 immunotherapy; **B** Immune responses of REEP4 to combination of CTLA4 inhibitors and PD1 inhibitors; **C** Immune responses of REEP4 to CTLA4 Immunotherapy; **D** TIDE score in different REEP4 subgroups; **E** T cell dysfunction score in different REEP4 subgroups; **F** T cell Exclusion score in different REEP4 subgroups; **G** Correlations between sensitivity of chemotherapy drugs with REEP4 expression.

We performed an analysis assessed the impact of REEP4 on the efficacy of cytotoxic T lymphocyte-associated antigen 4 (CTLA4) inhibitors and programmed cell death protein 1(PD-1) inhibitors in KIRC patients. Comparing the groups with high and low REEP4 expression levels, our findings demonstrated that REEP4 exhibited sensitivity to PD-1 inhibitors alone (p=0.0130) (Figure 6A), as well as to the combination of CTLA4 inhibitors and PD-1 inhibitors (p=0.0007) (Figure 6B). However, the use of CTLA4 inhibitors alone may not yield significant effectiveness (Figure 6C).

Subsequent to our analysis, we discovered a significant decrease in the TIDE score within the low expression group of REEP4 compared to the high expression group (p<0.0001) (Figure 6D). Additionally, the impact on T cell dysfunction was less pronounced in the low-expression group (p<0.0001) (Figure 6E). However, no notable disparity in the T-cell exclusion score was observed between the two groups (Figure 6F). These findings suggest that REEP4 plays a pivotal role in the effectiveness of immunotherapy in KIRC.

Moreover, the sensitivity to anticancer drugs based on REEP4 expression was assessed using the CellMiner database. We found that the expression of REEP4 was significantly positively correlated with sensitivity to quizartinib and SNS-314 drugs (Figure 6G).

## Discussion

Biomarkers serve as crucial cancer signatures and have applications in cancer screening, diagnosis, treatment, and prognosis. Detecting biomarkers enables early intervention, increases the likelihood of accurate personalized cancer therapy, and greatly advances cancer diagnosis and treatment 17. Owing to the metastatic potential of KIRC, it is imperative to identify a reliable tumor marker that can predict prognosis and clinical outcomes. Previous studies have reported significant associations between other REEP family members and cancer development. For instance, elevated levels of REEP3 have been found to increase circFAT1 expression, ultimately promoting the spread and infiltration of hepatocellular carcinoma cells 9. REEP6 polymorphisms have been shown to influence the stability of REEP6 mRNA, inhibiting apoptosis and stimulating colon cancer growth 18. However, in the case of REEP4, which is also a member of the REEP family, no prior report has detailed its association with cancer development. Compared to healthy tissues, tumors exhibit diverse transcriptomic profiles. Earlier studies have predominantly focused on identifying differentially expressed genes in tumors, leading to critical discoveries of biomarkers and therapeutic targets 19. Motivated by this, we conducted a pan-cancer analysis using the REEP4. Our findings indicate a significant upregulation of REEP4 expression in tumor tissues across various cancers when compared to normal tissues. This suggests that REEP4 may play a contributory role in tumor progression. Therefore, we investigated the association between REEP4 and KIRC.

Utilizing data from TCGA and GEO datasets, we procured comprehensive information encompassing both cancerous and normal tissues from numerous KIRC patients. Our analysis showed that REEP4 expression was significantly upregulated in KIRC tissues compared to that in normal tissues. Notably, this elevation in expression was positively correlated with the degree of malignancy of the tumor.

This observation aligns with previous findings where heightened REEPs expression was found to intensify lung cancer cell proliferation and metastasis, ultimately leading to diminished survival rates 10. To further determine the correlation between REEP4 expression and KIRC prognosis, we conducted COX regression analysis along with single and multifactorial assessments. The results revealed that KIRC cases with high REEP4 expression typically had a less favorable prognosis. Subsequently, we corroborated these analytical findings through an experimental validation. Using Western blot and RT-PCR techniques, we detected KIRC tissues obtained from the hospital, as well as renal cancer cells. These results were consistent with our previous analyses, which revealed significantly elevated levels of REEP4 expression in both KIRC tissues and renal cancer cells. This convergence between analytical and experimental data bolsters the robustness of our findings.

We conducted a comprehensive examination of the biological functions of REEP4, revealing that it is predominantly localized in the cytosol and nucleoplasm. Notably, REEP4 is strongly implicated in vital cellular processes, such as cell division and protein synthesis. This finding aligns with previous research, where Darshan et al. confirmed the requirement of REEP4 for high ER membrane curvature during mitosis 20. Additionally, Zhang et al. verified that modulating mitosis could be an effective strategy to prevent the progression of kidney cancer 21. Moreover, REEP4 is associated with the cell cycle. Qian et al. found that inhibiting cyclin D1 expression can effectively suppress kidney cancer growth 22, providing substantial evidence for the involvement of REEP4 in the development of KIRC. These findings were consistent with the results presented by Fan et al. 5. Carrying out further GSEA analysis, we found that REEP4 may play an important role in KIRC through signaling pathways, including chemokine, primary immunodeficiency, and T cell receptor pathways.

Cancer immunotherapy shows great potential for treating cancer by increasing the body's immune response and preventing immune evasion to eliminate tumors 23. This approach has demonstrated notable success in treating melanoma, lung cancer, and renal cell carcinoma 24. GSEA analysis revealed that REEP4 is associated with the T-cell receptor pathway. Thus, we conducted immune correlation analysis to discern the relationship between REEP4 and KIRC. Employing the ESTIMATE tool, we found that the tumor microenvironment has significant relevance in both the diagnosis and prognosis of KIRC. The level of immune cell infiltration within the tumor microenvironment, including B and T cells, NK cells, and DC, has a significant impact on patient survival and the effectiveness of immunotherapy across various cancer types, including melanoma 25. Through TIMER analysis, we established a positive correlation between REEP4 expression and immune cell infiltration in KIRC. We also found that REEP4 expression may be associated with activated CD4 T cells, effector memory CD4 T cells, etc. Correlation analysis between REEP4 and immune checkpoint genes revealed that REEP4 expression may be associated with checkpoint genes such as BTLA, CD244, and CD27. Moreover, by leveraging GSVA, a method based on Gene Set Enrichment (GSE) that estimates the variation in pathway activity within a sample population in an unsupervised manner 26, we demonstrated the capacity of REEP4 to regulate the immune response pathway in KIRC. To date, immunotherapeutic studies on related checkpoint genes and immune pathways in KIRC and studies on REEP4 and related immune targets are still lacking. These results provide new perspectives on REEP4 for immunotherapy in KIRC, revealing its potential targets and associated immune pathways. Thus, differential expression of REEP4 may contribute to tumor immunotherapy.

Over the past few years, immunosuppressive agents, including PD-1/programmed death-ligand 1 (PD-L1) inhibitors and CTLA4 inhibitors, have emerged as pivotal components in the treatment regimen for advanced or metastatic RCC 27. Subsequently, we evaluated the immunotherapeutic potential of REEP4 in KIRC using IPS and TIDE. The IPS demonstrated superior predictive capabilities for response to both anti-CTLA-4 and anti-PD-1 in two distinct validation cohorts 13. Our IPS findings provided compelling evidence that REEP4 expression may influence the therapeutic efficacy of PD-1 inhibitors, both as monotherapy and in combination with CTLA4 inhibitors, in KIRC patients. TIDE is a computational method designed to model the two primary mechanisms underlying tumor immune evasion. These mechanisms involve inducing T cell dysfunction in tumors characterized by high infiltration of cytotoxic T lymphocytes (CTL) and preventing T cell infiltration in tumors with low CTL levels. TIDE can be an effective tool for accurately predicting the prognosis of cancer patients undergoing first-line anti-PD1 or anti-CTLA4 therapy 28. Our analysis results demonstrated that patients exhibiting high expression levels of REEP4 tended to have lower TIDE and dysfunction scores. Furthermore, patients with elevated REEP4 expression may face an increased risk of immune evasion, and consequently experience reduced success in immunotherapeutic interventions. Furthermore, we used the CellMiner database to explore potential chemotherapeutic drugs that are sensitive to patients with high expression of REEP4 in KIRC 29. We found that the expression of REEP4 was significantly positively associated with sensitivity to quizartinib and SNS-314 drugs. This suggests that patients with high REEP4 expression may benefit from these drugs. However, it should be noted that the sensitivity of certain immunotherapeutic drugs, such as dasatinib and pluripotin, is negatively correlated with the expression level of REEP4. This suggests that these drugs may not be suitable for patients with KIRC with high REEP4 expression levels (Table S6). These insights underscore the potential impact of REEP4 on immunotherapeutic outcomes in KIRC patients.

## Conclusion

By integrating bioinformatics analysis with pertinent experimental validations, our study provides compelling evidence for the upregulation of REEP4 expression in KIRC, indicating a close association with the disease. This suggests the potential of REEP4 as a biomarker with significant influence on the prognosis and clinical treatment effects of KIRC. Our analysis showed that high expression of REEP4 affects the survival and immunotherapy outcomes of KIRC patients, suggesting that REEP4 has the potential to be a biomarker with a significant impact on the prognosis and clinical outcome of KIRC. However, our study is based on bioinformatics analysis, and further confirmation needs to be refined by biological or animal experiments. Moreover, given the higher expression observed in cases with distant metastases, it is worth exploring whether REEP4 contributes to the invasiveness of KIRC. In conclusion, our study explains the potential value of REEP4 as a marker for predicting the prognosis and immune response of KIRC and provides a novel idea for exploring therapeutic targets for KIRC.

## Supplementary Material

Supplementary tables.

## Abbreviations

KIRC: Kidney clear cell carcinoma
RCC: Renal cell carcinomas
REEPs: receptor expression-enhancing proteins
ROC: Receiver Operating Characteristic
GO: Gene Ontology
KEGG: Kyoto Encyclopedia of Genes and Genomes
GSEA: gene set enrichment analysis
BRCA: breast invasive carcinoma
CHOL: cholangiocarcinoma
LIHC: liver hepatocellular carcinoma
CTLA4: cytotoxic T lymphocyte-associated antigen 4
PD-1: programmed cell death protein 1
